# Advances in Natural Polymers: Extraction Methods and Applications

**DOI:** 10.3390/polym16131886

**Published:** 2024-07-01

**Authors:** Cornelia Vasile, Gabriel Aguirre-Álvarez, Xiao-Feng Sun

**Affiliations:** 1 Physical Chemistry of Polymers Department, Petru Poni Institute of Macromolecular Chemistry, Romanian Academy, 41A Gr. Ghica Voda Alley, 700487 Iași, Romania; 2 Instituto de Ciencias Agropecuarias, Universidad Autónoma del Estado de Hidalgo, Av. Universidad Km 1, Rancho Universitario, Tulancingo 43600, Hidalgo, Mexico; aguirre@uaeh.edu.mx; 3 School of Chemistry and Chemical Engineering, Northwestern Polytechnical University, Xi’an 710129, China; xf001sn@nwpu.edu.cn

Biomass-based alternatives for the manufacturing of bioplastic materials are important aspects of a more sustainable future; their physicochemical properties need to be able to compete with the existing market to establish them as a viable alternative. This Special Issue, aims to present recent modern trends and scientific results on natural polymers. Natural polymers normally occur in nature and can be obtained from a wide variety of sources including plants, animals, and microorganisms. They present special characteristics such as high biodegradability, good biocompatibility, and external-stimuli responsiveness have been widely applied in various engineering fields, including food packaging and drug delivery, and they can be modified by physical and chemical methods to obtain multifunctional materials, etc.

The aim of the Special Issue entitled “Advances in Natural Polymers: Extraction—Methods and Applications” is to collect the latest original research studies on biomass and natural polymers, which are promising valuable sources for the creation of a sustainable and green circular economy, they are environmentally friendly, and also can facilitate the development of nanosized high-performance materials. Authors provided valuable contributions which collectively built a successful issue that will give to its readers an overview of the state-of-the-art activities and the future perspectives in this field.

The aim of this Special Issue is to advance our understanding of fundamental and technological aspects of the extraction of natural polymers and active compounds and their applications in various fields. Biomass has a complex chemical structure and a variety of bioactivities. Biomass valorization is a very interesting topic, because depending on its nature, cultivation conditions, and processing methods (including extraction), its composition, properties, and biological activities, such as anti-oxidative, hypoglycemic, hypolipidemic, immune regulation, and anti-tumor activities, are variable. This editorial provides brief overview on the recent developments in this field, the gaps in knowledge and how this Special Issue addressed those gaps, and it ends with a primary focus on the future research that should be considered. Submissions of original research articles or reviews from an extensive range of expertise in the wide-ranging field of natural polymers were both welcome. Natural polymers are mainly classified into the following groups: polysaccharides (cellulose, hemicelluloses, pectin, dextran, pullulan, starch, chitin, chitosan, alginate, hyaluronic acid, xanthan, guar gum, etc.), peptides/proteins (collagen, gelatin, casein, albumin, etc.) and polynucleotides (DNA and RNA), lignin, polyisoprenes, and polyesters. Natural polymers are gaining interest among the research community both as renewable sources, lack of toxicity, low cost, and bioactive compounds as attractive ingredients for the food industry (packaging, dietary supplements, food supplements, etc.), biomedicine, and cosmetics (skin repair and regeneration, bone tissue engineering, moisture agents, greater cellular attachment and matrix deposition, mechanical stability, drug carriers in anticancer therapy, nanocarriers, high compatibility with the extracellular matrix, high bioavailability, safety, anti-inflammatory, antimicrobial and antioxidant activity, film formation, nutraceutical beverages, hydration of the skin, etc). [1,2,3,4].

This Reprint in *Polymers* which belongs to the Polymer Applications section, has a highly modern and interesting purpose because it contains publications regarding new aspects of progress/extraction and applications of some natural polymers from various sources. Also, agrowaste or byproducts containing valuable natural components have been used as new sources of raw materials and active agents to replace synthetic plastics known by their environmental and human health impacts. Both research papers and reviews are focused on green, eco-friendly, and easily scalable processes, with a view to facilitate a sustainable and circular economy. Some research papers deal with new extraction procedures to obtain celullose or lignin and the recycling of biomass into high-value-added materials, as well as important developments in research and technology, which have also been recorded via the study of some proteins and different polysaccharides such as chitosan, tea polysaccharides, hyaluronan, reducing sugars in foods to obtain silver nanoparticles and the valorization of some by-products such as Nopal Mucilage and milk whey hydrolysates.

This Reprint is addressed to experts in the large-scale development/valorization of different natural resources into high-value materials, engineers, researchers, and PhD students who may be performing their studies using the modern techniques used in the published papers.
